# Genome-Wide Identification and Stage-Specific Expression Profile Analysis Reveal the Function of Ribosomal Proteins for Oogenesis of *Spodoptera litura*

**DOI:** 10.3389/fphys.2022.943205

**Published:** 2022-06-23

**Authors:** Ranran Sun, Jin Liu, Yuanhao Xu, Liwei Jiang, Yun Li, Guohua Zhong, Xin Yi

**Affiliations:** ^1^ Key Laboratory of Crop Integrated Pest Management in South China, Ministry of Agriculture, South China Agricultural University, Guangzhou, China; ^2^ Key Laboratory of Natural Pesticide and Chemical Biology, Ministry of Education, South China Agricultural University, Guangzhou, China

**Keywords:** *Spodoptera litura*, ribosomal proteins, oogenesis, phylogenetic relationships, gene expression

## Abstract

Ribosomal proteins (Rps) are indispensable in ribosome biogenesis and protein synthesis, which tightly correlate with cell growth and proliferation in different physiological processes across species. Up to now, genes coding for Rps have been identified and studied in many species, however, their information still remains elusive in many insect species, especially in *Spodoptera litura*. In this study, 81 *Rp* genes were identified from *S. litura* genome and were mapped to their positions on the chromosomes. In addition, their physical and chemical properties, gene structure, phylogenetic relationships, targeted microRNAs were also analyzed. Gene ontology analysis disclosed that *Rp* genes were closely associated with processes related to ribosome biosynthesis, proteins translation processing, molecular binding activities. The quantitative real-time PCR (qRT-PCR) revealed expression profiles of *Rp* genes varied in different stages of oogenesis, and found that most *Rp* genes accumulated in previtellogenesis stage. This study described the comprehensive genome-wide analysis of *Rp* gene family in agricultural pests, which provided foundation for further characterizing the roles of Rps in oogenesis of insects, and some *Rp* genes may further serve as targets for innovative pest control.

## Introduction

In agricultural production, pests always directly and indirectly cause losses to crop production and threaten livelihoods due to their high fecundity, environmental adaptability, short life cycle or increased insecticide resistance (Cheng et al., 2017; Du et al., 2017). Oviposition depended on oocyte maturation during oogenesis is an important phase for successful reproduction of insects (Wang et al., 2021). Therefore, screening and application of efficient and environmentally friendly chemicals targeting oogenesis could be an important approach for pest management. Many factors, including vitellogenin (Vg), the biosynthesis of juvenile hormone (JH) and ecdysone as well as insulin associated with nutritional metabolism were known to play vital roles in the regulation of oogenesis in most insect species (Smykal and Raikhel, 2015; Roy et al., 2018; Song et al., 2018). For example, Vg was synthesized in fat body and taken up by maturing oocytes for egg production and female reproduction (Wu et al., 2016; Zhu et al., 2020). Egg 80 protein is one of the components in eggshell, which is secreted by follicle cells during the late vitellogenesis stage to early choriogenins stage, and the absence of egg 80 influences the integrity of eggshell and causes the collapse in *Bombyx mori* (Xu et al., 2011). Muskelin is up-regulated during oocyte maturation, which is required for timely nurse cell nuclei clearing from mature egg chambers (Kronja et al., 2014). The nutrient sensors mediated by insulin/IGF signaling (IIS) and target of rapamycin (TOR) could promote vitellogenesis in an indirect manner *via* activating JH biosynthesis in *Periplaneta americana* (Zhu et al., 2020). 20-hydroxyecdysone (20E, the active form of ecdysone) signaling was suppressed and blocked during ovarian development in *Colaphellus bowringi* under the long-day condition (Guo et al., 2021). Pancreatic lipase-related protein 2 gene was required for oocyte maturation and development in brown planthopper, *Nilaparvata lugens* (Wang et al., 2021). There is no doubt that the regulatory mechanism of oocyte maturation during oogenesis is complex and multi-factor determined, in addition to these characterized genes, many other unknown genes may be also involved in this reproductive process.

Ribosomes have been classically viewed as static, homogeneous molecular machines capable of indiscriminate translation of the entire population of mRNAs within cells (Mageeney and Ware, 2019), which are at the critical junction of the genotype-phenotype relation in all species (Polymenis, 2020). Ribosomal proteins (Rps) are structural and noncatalytic components of ribosomes (Lee et al., 2018), and Rps from the small and large subunits are named as RPS and RPL, respectively (Kuang et al., 2020). All the core and tissue-specific RPS and RPL compositions within ribosomes could contribute to ribosome heterogeneity and functional variability (Kronja et al., 2014; Gershman et al., 2020). Moreover, some studies have suggested that Rps are essential for reproduction and oocyte maturation. For example, in *Xenopus*, the synthesis of Rps during oogenesis contributes to the production of a maternal ribosome pool that is sufficient to support protein synthesis throughout embryogenesis (Hyman and Wormington, 1988). In mice, loss of RPS26 arrests oocyte growth and causes premature ovarian failure (Liu et al., 2018). In *Aedes aegypti* mosquito, depletion of RPL26, RPS6 and RPL32 could significantly reduce fecundity (Estep et al., 2016; Wang et al., 2017). In *Drosophila melanogaster*, RPS5b is the most highly expressed Rp in ovaries in contrast to its paralog (RPS5a), and loss of RPS5a in the germline does not cause a germline phenotype, however, loss of RPS5b could result in a mid-oogenesis defect that is further exacerbated when RPS5a is depleted in a RPS5b mutant background (Kong et al., 2019). However, the systematic assessment of Rps in reproduction function has not been well studied, especially in agricultural pests.

The tobacco cutworm, *Spodoptera litura* (Lepidoptera, Noctuidae), is an important polyphagous pest. The larvae of *S. litura* can feed on over 100 species of crop plants and cause heavy yield losses, and the high fecundity and short life cycle of this pest under tropical conditions could result in a high rate of population increase and possible population outbreaks (Cheng et al., 2017). Thus, *S. litura* has been extensively studied as a representative pest in agricultural ecosystem. The ovary of *S. litura* belongs to polytrophic meroistic ovarian, and the oogenesis can be sequentially subdivided into three developmental periods: previtellogenesis, vitellogenesis and choriogenesis (Sun et al., 2022), which may require many genes for the synthesis of those functional proteins. The released genome of *S. litura* could provide important resources for further molecular researches of this species (assembly ASM270686v3) (Cheng et al., 2017). Here, *S. litura Rp* (*SlRp*) genes were identified, and the gene chromosomal location, exon/intron and conversed motif structural analysis were studied to show the sequence characteristics of *SlRps*. The phylogenetic tree was constructed to enrich the evolutionary relationship of *SlRps* with other representative insects. Finally, the stage-specific expression patterns of *Rp* genes in three developmental periods of oogenesis were analyzed. Collectively, our research provided a novel insight underlying the roles of Rps in oogenesis in *S. litura* and reference resources in developing environmentally friendly insecticides.

## Materials and Methods

### Insect Rearing

The larvae of *S. litura* were reared on artificial diet at 25 ± 1°C with 60%–70% relative humidity, and a 16 h light/8 h dark photoperiod in the laboratory. For all the adults feeding, cotton balls soaked in 10% honey were provided for nutrition supply.

### Identification of *Rp* Genes From Different Insect Species

Rps are highly conserved even between vertebrates and invertebrates. Therefore, protein sequences of Rps from 6 insect species (*S. litura*, *B. mori*, *S. frugiperda*, *Helicoverpa armigera*, *Plutella xylostella* and *D. melanogaster*) were obtained from their genome databases in NCBI (National Center for Biotechnology Information) using Basic Local Alignment Search Tool (blast2.0) searched with Human Rps as the query sequences. After the Rps were annotated using Swiss-Prot database, the identified Rps were named and listed in Supplementary Datasheet S1 according to the principle described in Human and *D. melanogaster* (Yoshihama et al., 2002). Briefly, (I) if there are multiple gene_ids corresponding to the same transcript, these genes would be uniformly classified into the same transcript, and one of the gene_ids was selected to be registered in Supplementary Datasheet S1; (II) if one gene had multiple transcripts, the longest transcript was represented for the gene in Supplementary Datasheet S1; (III) the multi-copy genes were distinguished by adding a, b, c, d, etc., following gene_name, and the gene_names were showed in Supplementary Datasheet S1.

### Phylogenetic Tree Construction

For the construction of phylogenetic trees, the amino acid sequences of Rps from 6 insect species (*S. litura, B. mori, S. frugiperda, H. armigera, P. xylostella* and *D. melanogaster*) were aligned using ClustalW with the default parameters. The neighbour-joining (NJ) phylogenetic trees were generated using MEGA X (https://www.megasoftware.net/) with 1000 bootstrap replications and visualized using iTOL online website (https://itol.embl.de/). Besides, the maximum-likelihood (ML) phylogenetic tree was reconstructed by IQ-TREE to shown the evolutionary relationship of Rps in *S. litura.*

### Chromosomal Mapping and Duplication Analysis of *S. litura Rp* Genes

Every *SlRp* was matched with the chromosomes of *S. litura* based on the genome annotations. MapGene2Chrome (http://mg2c.iask.in/mg2c_v2.0/) was used to draft the map. MCScanX (default parameters) was used to examine duplicated genes with default parameters.

### The Structure and Gene Ontology Function Analyses of *S. litura Rp* Genes

The *SlRp* gene structures were analyzed and showed by Tbtools (V 1.098669) software with default parameters (Chen et al., 2020). The conserved structural domains of *Sl*Rps were predicted in smart database (https://smart.embl-heidelberg.de) and conserved domain database (CDD) in NCBI (https://www.ncbi.nlm.nih.gov/Structure/cdd/cdd.shtml). In addition, the Gene ontology (GO) function annotation was carried out using a free online platform HYPERLINK (http://eggnog-mapper.embl.de/), and Tbtools (V 1.098669) software was used for GO enrichment analysis (Chen et al., 2020).

### *Sl*Rp Protein Properties and Prediction of Putative microRNAs Targeting *S. litura Rp* Genes

In the current study, ProtParam (http://web.expasy.org/protparam/) was used for the prediction of the physical and chemical features of those *Sl*Rps. The online subcellular localization tool Cell-PLoc 2.0 (http://www.csbio.sjtu.edu.cn/bioinf/Cell-PLoc-2/) was used to perform subcellular localization prediction for these 81 *Sl*Rps. In addition, for miRNA target prediction, mature *S. litura* miRNA sequences were obtained from insectbase2.0 (http://v2.insect-genome.com/), the *SlRp* 3'UTR sequence were obtained from *S. litura* genome database (https://www.ncbi.nlm.nih.gov/genome/?term=Spodoptera+litura), and miRanda algorithm (version 3.3a) (http://www.bioinformatics.com.cn/local_miranda_miRNA_target_prediction_120) was used to predict targeted miRNAs with default parameters (score = 140, energy = −1).

### Expression Profiles Analysis

To detect the stage-specific expression patterns of *SlRps*, we collected the ovarian fragments of previtellogenesis stage (PS), vitellogenesis stage (VS.) and choriogenesis stage (CS) from female adults (2rd day after eclosion) based on the presence and positions of nurse cells (Sun et al., 2022). The total RNA was extracted by RNAiso Plus (TaKaRa, Japan), and the first-strand cDNA was synthesized using PrimeScript™ RT reagent Kit with gDNA Eraser (Perfect Real Time) according to the manufacturer’s instructions (Takara Biomedical Technology, Beijing, China). qPCR was performed with the iTaq^TM^ SYBR^®^ Green Supermix (Bio-Rad, Hercules, CA) according to the manufacturer’s protocol, and all qPCR reactions were conducted on a CFX96 System (Bio-Rad, United States). The primers were listed in Supplementary Table S1. *GAPDH* was chosen as a reference gene, and the expression levels of target genes are relative to *GAPDH* for standardization. The data from three independent experiments were obtained by relative expression levels and were calculated using the 2^−△△C^_T_ method.

### Data Analysis

The statistical analyses were performed using GraphPad Prism 8 and one-way ANOVA were employed to check the significant differences. Differences were considered significant at *p* value < 0.05, and the data are expressed as mean ± SEM (standard error of mean).

## Results

### Identification and Characteristics of *S. litura Rp* Genes

We identified a total of 81 *SlRp g*enes from *S. litura* genome in NCBI, including 34 encoding RPS and 47 encoding RPL. The GenBank accession number, chromosome location, number of exons, gene length (bp) for the complete set of *Rp* genes were showed in Supplementary Datasheet S2. All the 81 *Rp* genes have complete open reading frames (ORFs) and gene sequences. The average size of the genes from the transcription start site is ∼2.40 kb, among which, *RPL4* is the largest (∼11.33 kb), whereas *RPL24a* is the smallest (∼0.52 kb). In addition, each gene has an average of 4.15 exons, ranging from 1 (*RPL24a*, *RPL28* and *RPS3Ab*) to 10 (*RPL4*).

### *S. litura* Rps Properties and Subcellular Location

The biochemical characteristics of *Sl*Rps were showed in Supplementary Datasheet S3. The protein molecular weight (MW) ranged from ∼6.36 kDa (RPL39) to ∼53.21 kDa (RPL4), and isoelectric points (pI) varied from 4.17 (RPLP1) to 12.55 (RPL39), and most (*n* = 75, 92.59%) of them were in range of 9.39–12.55 with a few exceptions, such as RPLP2 has a very low pI of 4.17. In addition, we found that positively charged amino acids like Arg and Lys were enriched in some Rps. However, RPL39 has no negatively charged residues. The Grand Average of Hydrophobicity (GRAVY) indices were below the value of zero for all the 81 *Sl*Rps, suggesting the hydrophobic nature of *Sl*Rps. Besides, the subcellular location prediction revealed that *Sl*Rps were mainly located in nucleus (*n* = 51, 62.96%), mitochondrion (*n* = 28, 34.57%), cytoplasm (*n* = 15, 18.52%), extracell (*n* = 6, 7.41%) and endoplasmic reticulum (*n* = 1, 1.23%).

### Chromosomal Distribution and Synteny Analysis of *S. litura Rp* Genes

**FIGURE 1:**
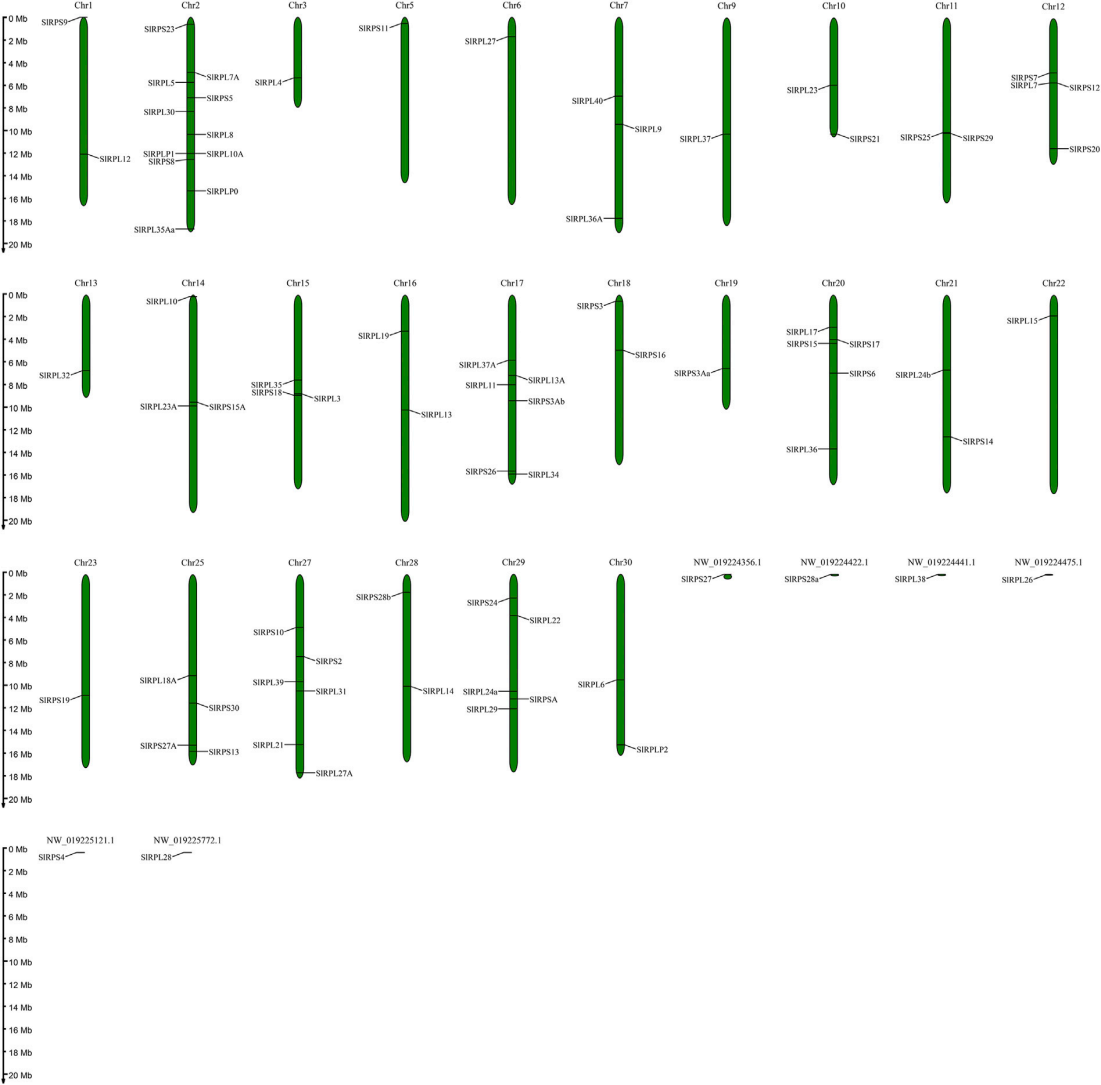
Chromosomal distribution of *SlRp* genes.

**FIGURE 2:**
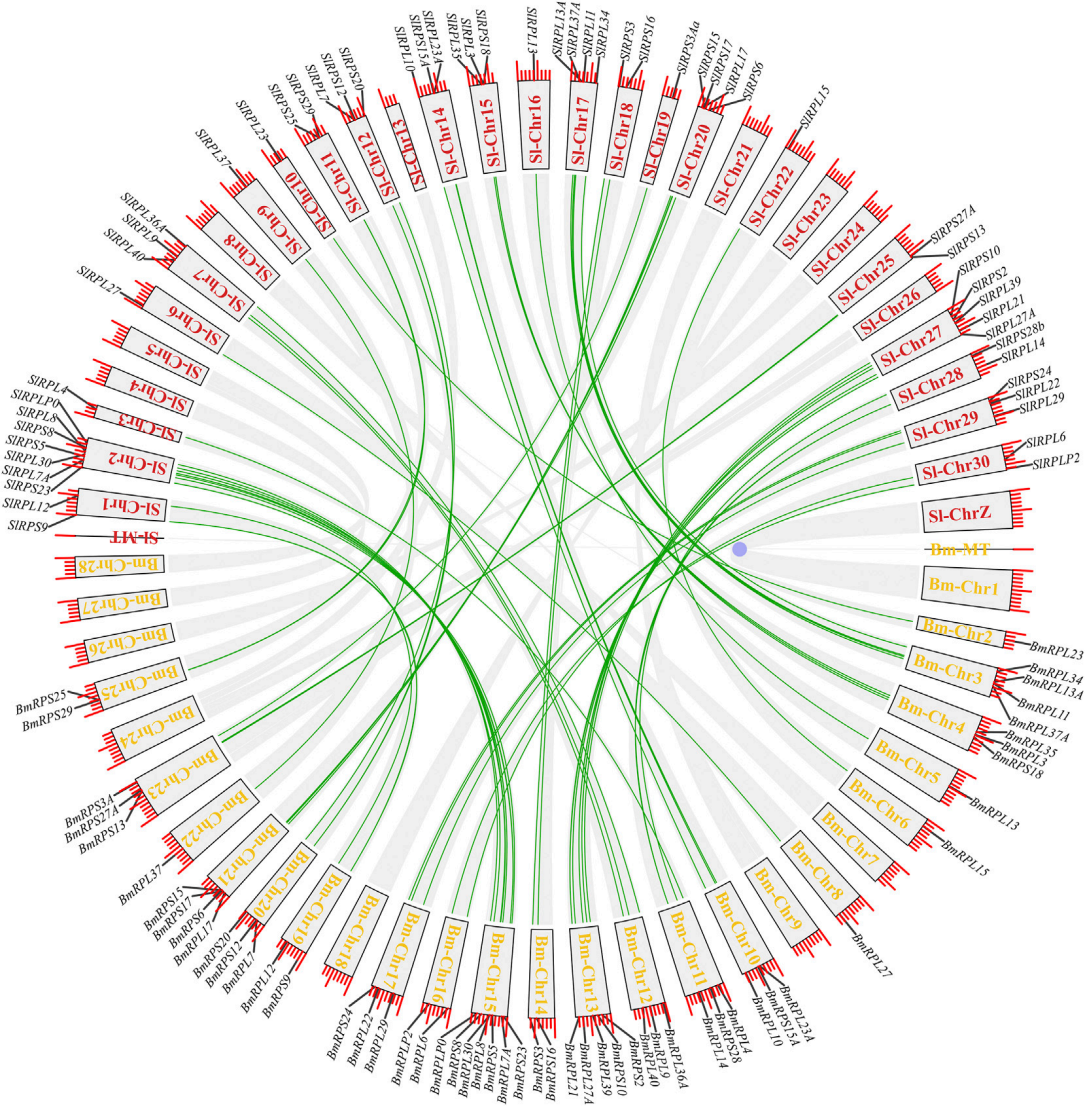
Synteny analysis of *Rp* genes in *S. litura* and *B. mori*. The red and yellow fronts in the blocks represented the chromosome labels in *S. litura* (*Sl*) and *B. mori* (*Bm*), respectively, and the green lines refer to the orthologous *Rp* genes in *S. litura* and *B. mori*.

According to genome annotation information, 81 *Rp* genes were assigned to the linkage maps of *S. litura* genome (Figure 1). Among them, 75 *Rp* genes were found to be irregularly distributed on 26 chromosome, and 11 *Rp* genes (∼14.67%) were present on chromosome 2, while no *Rp* genes were present on chromosome 4, 8, 24, 26 and chromosome Z. In addition, 6 *Rp* genes were localized on unassembled genomic scaffolds (Ding et al., 2021). To further explore the evolutionary mechanism of the *SlRp* gene family, we constructed a syntenic map of *S. litura* associated with Lepidoptera model insect, *B. mori*, as showed in Figure 2. A total of 54 syntenic orthologous gene pairs were identified between *S. litura* and *B. mori*, indicating that *S. litura* and *B. mori* shared a close relationship. And we found that one *SlRp* gene could only correspond to one gene in these syntenic orthologous gene pairs. Then, for further evolutionary studies, the divergence time of *Rp* gene pairs was calculated in the two varieties (Supplementary Datasheet S4), and the divergence time started 84.90 Mya to 12.22 Mya between *S. litura* and *B. mori*. Additionally, Ka/Ks (the ratio of nonsynonymous substitution to synonymous substitution) value < 1 indicates that a gene pair has experienced negative selection, whereas Ka/Ks > 1 indicates positive selection, and Ka/Ks = 1 indicates neutral selection (Cao et al., 2022). Our results showed that the Ka/Ks ratio varied from 0 to 0.0956, suggesting the evolution of *Rp* genes between *S. litura* and *B. mori* has experienced purify selection.

### Functional Annotation of *S. litura* Rps

**FIGURE 3:**
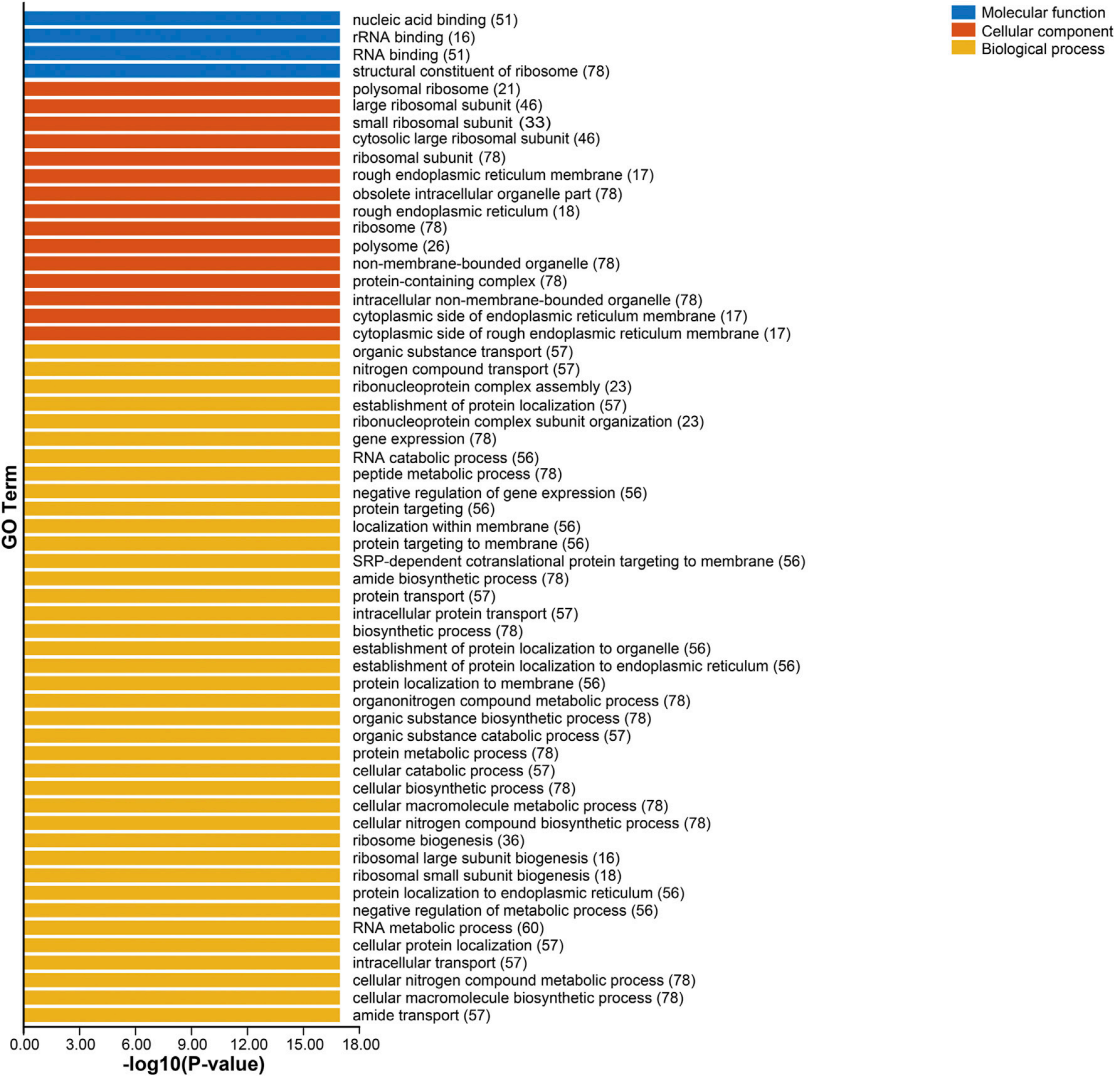
The GO enrichment terms of *Sl*Rps.

To further understand the functions of *Sl*Rps, we implemented gene ontology (GO) enrichment analysis based on three classes, biological process (BP), molecular function (MF), and cellular component (CC). The GO enrichment terms were showed in Supplementary Datasheet S5 and Figure 3, and GO-MF enrichment found that nucleic acid binding (GO:0003676, *n* = 51), rRNA binding (GO:0019843, *n* = 16) and RNA binding (GO:0003723, *n* = 51) were the main molecular functions for *Sl*Rps. The GO-CC enrichment discovered that most *Sl*Rps were important constituents of ribosome, rough endoplasmic reticulum and other intracellular organelle parts, including, large ribosomal subunit (GO:0015934, *n* = 46), small ribosomal subunit (GO:0015935, *n* = 33), rough endoplasmic reticulum membrane (GO:0030867, *n* = 17) and obsolete intracellular organelle part (GO:0044446, *n* = 78). Likewise, the GO-BP enrichment results suggested that *Sl*Rps were mainly participated in organic substance transport (GO:0071702, *n* = 57), nitrogen compound transport (GO:0071705, *n* = 57), ribonucleoprotein complex assembly (GO:0022618, *n* = 23), establishment of protein localization (GO:0045184, *n* = 57), RNA catabolic process (GO:0006401, *n* = 56) and peptide metabolic process (GO:0006518, *n* = 78). Overall, the GO enrichment analysis revealed the important roles of *Sl*Rps in ribosome biosynthesis, proteins translation processing, and molecule binding activity.

### Gene Structure and Evolutionary Relationships of *S. litura Rp* Genes

**FIGURE 4:**
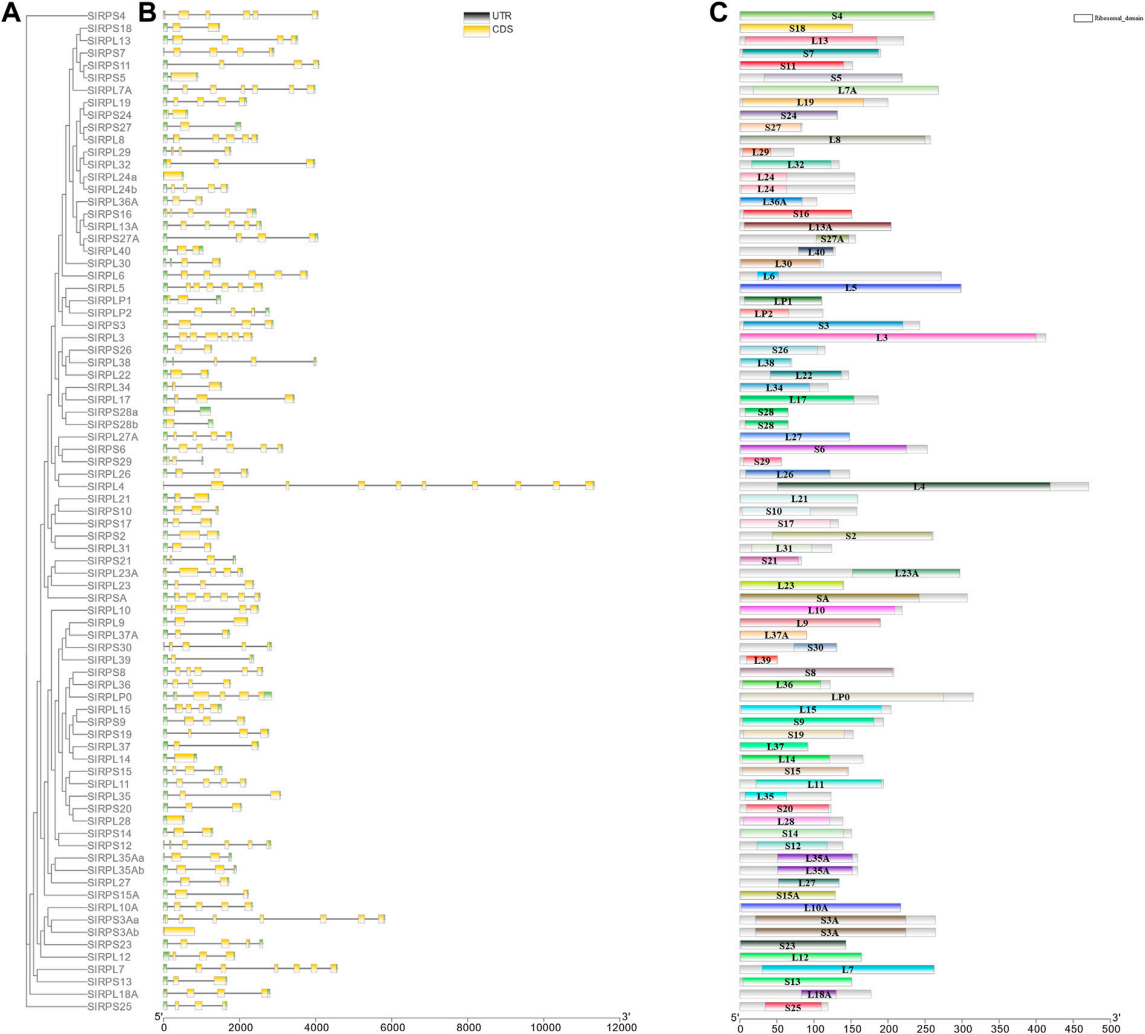
Phylogenetic relationship, motifs analysis, and gene structure of *SlRp* genes. **(A)**, the ML phylogenetic tree based on *Sl*Rps; **(B)**, Exon-intron structure of *SlRp* genes. Blackish-grey lines indicate introns and green boxes indicate UTR regions, and yellow rectangles indicate the position of *SlRp* CDS domains; **(C)**, Conserved domains of *Sl*Rps were predicted in smart database, and different-colored squares represent different domains of Rps.

To further understand the features of *SlRp* genes, we investigated the intron/exon configuration, GC-content and 5’and 3’ untranslated regions according to the phylogenetic ML tree with *SlRp* sequences (Figure 4A). Analysis of genomic DNA sequences showed that the number of introns changed from 0 to 9, the number of exons of *SlRps* ranged from 1 to 10, and the majority of *SlRp* genes (70.37%, *n* = 57) had high GC-content (Figure 4B and Supplementary Datasheet S2), for example, *RPS21*, *RPLP2* and *RPSA* hold 61.91%, 60.17% and 59.95% GC-content in CDS, respectively. Moreover, we also found that a few genes are multi-copy genes, which have the same intron/exon pattern, such as, *RPS28a* and *RPS28b*; *RPL35Aa* and *RPL35Ab*. However, *RPL24a* and *RPL24b*; *RPL3Aa* and *RPL3Ab* had different intron/exon patterns (Figures 4B,C). We examined the full-length protein sequences of 81 *Sl*Rps to recognize their conserved domains and found that there was no high similarity among different proteins (Figure 4C), suggesting the functional diversity of Rps in *S. litura*.

### Sequence Alignment and Phylogenetic Analysis

To understand the evolutionary relationships of Rps among *S. litura*, *B. mori*, *S. frugiperda*, *H. armigera*, *P. xylostella*, and *D. melanogaster*, phylogenetic analysis and NJ tree construction among 231 Rp sequences (81 *Sl*RPs, 84 *Bm*RPs, 88 *Sf*RPs, 81 *Ha*RPs, 83 *Px*RPs and 81 *Dm*RPs) (Supplementary Datasheet S1) were presented in Supplementary Figure S1. The phylogenetic tree showed that these Rps could be classified into eight distinct branches (clade I∼VIII), which contained 6, 8, 13, 15, 9, 9, 9 and 12 *Rp* genes, respectively (Supplementary Datasheet S6). In general, different proteins translated by the same gene from the same species were clustered together, such as *Sl*RPL35Aa and *Sl*RPL35Ab (clade I), *Sf*RPS7a and *Sf*RPS7b (clade I). However, there were also different cases, such as *Sf*RPL17a and *Sf*RPL17b (clade II), *Sl*RPL7Aa and *Sl*RPL7Ab (clade III). A few genes have different gene copy number (0–5) among different species, suggesting that *Rp* genes possibly expanded and diversified after the radiation of these different species, such as, *RPSA* (*n* = 0) in *S. frugiperda*, *RPL40* (n = 5) in *H. armigera* (Supplementary Datasheet S6).

### Analysis of miRNA Targeting *S. litura Rp* Genes

Increasing evidences have found that miRNA-mediated regulation is important in insect oogenesis (Li et al., 2022). Here, we predicted 247 putative miRNAs targeting at 38 *SlRp* genes, as shown in Supplementary Datasheet S7. We found that there were three types of miRNA targeting *SlRp* genes, the first type was that one miRNA (*n* = 100) could target at two or more *Rp* genes, such as, mir-137-2 targeted at *RPSA* and *RPS14* (Supplementary Figure S2A); another one was that two or more miRNA targeted at one *Rp* gene (*n* = 27), such as, mir-137-2, mir-10460-1, mir-2491, mir-13a-1, mir-10480-1 and mir-3529 targeted at only *RPSA* (Supplementary Figure S2B). The last type was that one miRNA only targeted at one *Rp* gene (*n* = 1), such as, mir-467g targeted at *RPS25* (Supplementary Figure S2C). These miRNA-genes regulatory networks of *Sl*Rps might provide important clues for their functional roles.

### Expression Profile of *S. litura Rp* Genes in Ovary During Oogenesis

**FIGURE 5:**
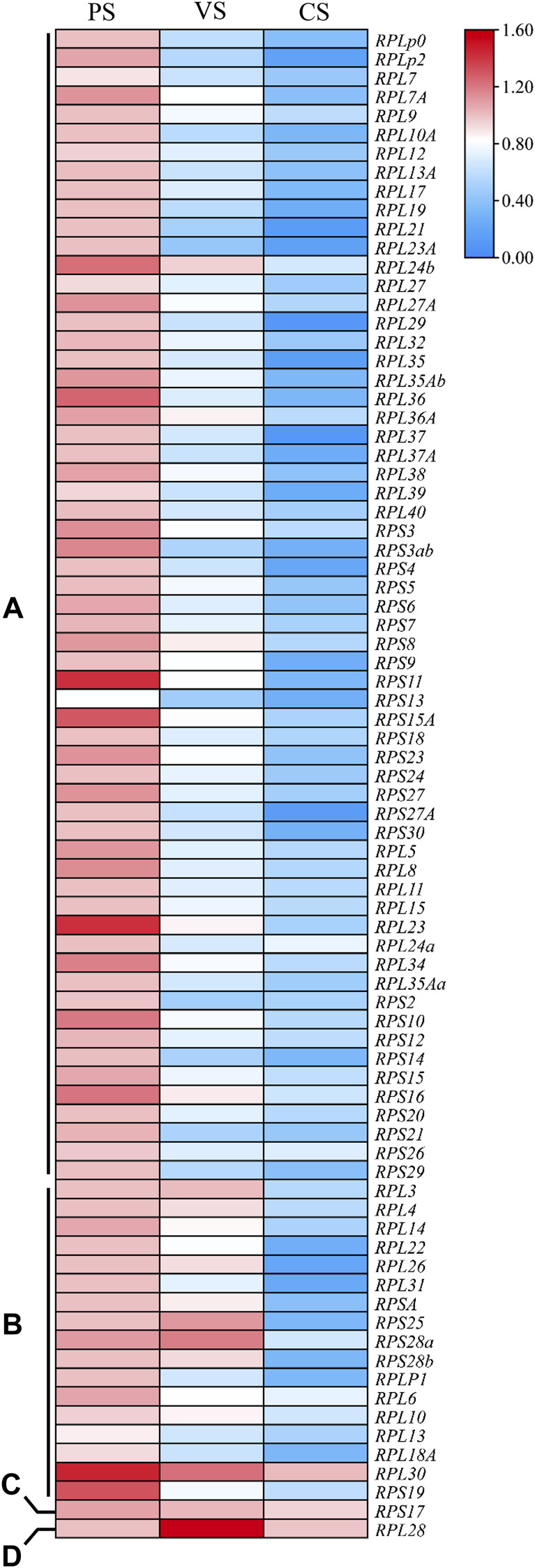
The heatmap for the expression patterns of *Rp* genes in oogenesis of *S. litura*, including previtellogenesis stage (PS), vitellogenesis stage (VS.) and choriogenesis stage (CS). **(A–D)** represented four different expression types of *Rp* genes among these three stages. The mean expression values were calculated from three independent biological replicates, and significance was determined using one-way ANOVA. *p* value < 0.05 represents significant difference. The heatmap was performed using Tbtools (V 1.098669) software based on the relative mRNA expression level of *SlR_p_* genes.

Gene expression profiles are closely related to gene biological functions. To understand the roles of *Rp* genes in oogenesis of *S. litura*, the stage-specific expression levels of 81 *SlRp* genes in PS, VS and CS were investigated by using qRT-PCR. As showed in Figure 5, we found that the expression levels of *SlRp* genes at three stages of oogenesis varied widely and could be classified into four types. Most *SlRp* genes (*n* = 62, 76.54%) were accumulated in PS, such as, *RPLP0* and *RPLP2* (Figure 5A). And the mRNA expression levels of 17 *SlRp* genes (12.35%) showed high expression at PS and VS. (Figure 5B). We also noted *RPS17* showed no significant difference among these three stages (Figure 5C), and *RPL28* showed higher expression level at VS than those at PS and CS (Figure 5D). These results highlighted that *SlRps* genes were involved in specific physiological functions related to oogenesis, especially in previtellogenesis development.

## Discussion

High fertility is an important physiological basis for the population outbreak of pests, which could drastically reduce crop yield (McFarlane et al., 2018). Rps are indispensable in ribosome biogenesis and protein synthesis, which has been suggested to play vital roles in insect fertility (Saha et al., 2017). The rapid development of genome sequencing and bioinformatics have increased the availability of complete sets of Rps in a wide range of species, including *Arabidopsis* (Barakat et al., 2001), rice (Saha et al., 2017), human (Yoshihama et al., 2002) and *Oreochromis niloticus* (Kuang et al., 2020). A systematic identification and characterization of insect Rps could guide the detailed functional study of these proteins. However, so far, genes coding for Rps have not yet been studied extensively in insects, and even less so in *S. litura*.

In this work, a total of 81 *Rp* genes were retrieved from *S. litura* genome databases. In total, 75 *SlRp* genes were found to be irregularly distributed on 26 chromosome, and 6 *SlRp* genes were localized on unassembled genomic scaffolds. Previous studies showed that gene organization is critical for the evolution of multiple gene families (Xu et al., 2012). As shown in Figure 4 and Supplementary Datasheet S2, we found that *SlRp* genes had different amounts of introns and exons, intimating these genes exhibited a high degree of complexity in structure and biological functions (Song et al., 2015; Yang et al., 2022). Based on the multiple sequence alignments, we found that the Ka/Ks ratios of all *SlRp* genes were smaller than 1, indicating that most *SlRp* genes have experienced extensive purifying selection (Supplementary Datasheet S4) (Cao et al., 2022). In addition, when it comes to the physicochemical properties of proteins, we found that positively charged residues (including Arg and Lys) were enriched in most *Sl*Rps, which might due to that positively charged amino acids play significant roles in electrostatic protein-RNA interactions within the ribosomal complex (Law et al., 2006; Takakura et al., 2016). It has reported that Rps are expected to have similar isoelectric pH ranges to facilitate molecular interactions (Saha et al., 2017). In silkworm, acidic RP p1 and p2 were required for functional protein binding to the GTPase-associated domain of 28S rRNA (Shimizu et al., 2002). In this study, acidic RPLP0, RPLP1 and RPLP2 proteins with low isoelectric points might be beneficial for minimize non-specific interactions. In terms of subcellular localization, we noted that most *Sl*Rps (62.96%) were located in nucleus (Supplementary Datasheet S3), which might be due to that ribosome biogenesis mainly takes place in the nucleus, and Rps associate with rRNAs through a highly complex and coordinated process to form ribosome subunits (de la Cruz et al., 2015). These characteristics suggested the key roles of Rps in modulating the structure and function of rRNAs (Warner and McIntosh, 2009). Some Rps hold multiple subcellular localizations, which could imply their different cellular functions. For instance, the subcellular distribution of fly eRPL22 changes during the course of sperm maturation. In mitotic stages, eRPL22 is found within nucleoli and the cytoplasm, endorsing the role of eRPL22 in ribosomal function in spermatogonia during the transit amplification stage of spermatogenesis. In the meanwhile, in meiotic spermatocytes, eRPL22 is found in the nucleoplasm of germ cells, suggesting the ribosomal role for eRPL22 (Mageeney et al., 2018). Indeed, in our study, a total of 18 Rps held two or more subcellular localizations, including RPL21 localized at cytoplasm, extracell, mitochondrion and nucleus, while RPL14 located at mitochondrion and nucleus, implying the functional diversities of these Rps in *S. litura*.

Polytrophic meroistic ovaries could be subdivided into three developmental periods: previtellogenesis, vitellogenesis and choriogenesis (Zhang et al., 2017). Previtellogenic development represents the formation of oocytes from oogonial stem cells by mitosis and meiosis (McLaughlin and Bratu, 2015). Vitellogenesis is the process of accumulating Vg and other biomaterials into growing oocytes (Sappington and Raikhel, 1998; Tang et al., 2020). After oocytes are fully grown, they are coated with chorion by follicular epithelium to become “eggs” in the proximal part of ovarioles (Al Baki et al., 2019). Given the different functions of different oogenesis stages for egg maturation, cells may need different Rps for protein processing. It has reported that tissue-specific or developmental stage-specific expression profiles of Rps would determine their different roles in regulating biological process (Kuang et al., 2020). The levels of ribosome biosynthesis during early oogenesis are strictly regulated and shockingly dynamic (Blatt et al., 2020). In this study, qRT-PCR analysis revealed that 62 *SlRp* genes were highly expressed in PC (Figure 5A), which was consistent with our observation that stem cells may be particularly sensitive to perturbations in ribosome biogenesis. Likewise, *Drosophila* germline stem cells also have a specific requirement for ribosome biogenesis, which varied greatly over the courses of germline stem cell differentiation (Sanchez et al., 2016). miRNAs, a group of single-stranded non-coding RNAs (∼21 nt long), could negatively regulate gene expression by cleavage of cognate mRNAs or translational blockage at the posttranscriptional level (Yogindran and Rajam, 2016; Shcherbata, 2018). In current work, we predicted 247 putative miRNAs targeting at 31 *SlRp* genes (Supplementary Datasheet S7). Twenty-two of these *Rp* genes were highly expressed in PS (Figure 5A), suggesting the key regulations of *Rp* genes and their related miRNAs in cell differentiation and subsequent egg mature. However, the role of miRNAs in ribosomal biogenesis is still uncertain, for instance, the mechanism of miRNA-10a to bind 5’ UTR of RP mRNAs and influence their translation (Orom et al., 2008), and the regulatory role of miR-542-3p in promoting ribosomal stress by targeting Rps and to reduce ribosomal RNA and protein synthesis (Connolly et al., 2018).

## Conclusion

In summary, based on *S. litura* genome, we identified 81 *SlRp* genes, and assessed their structure, chromosomal location, phylogeny, and related miRNAs. By analyzing the differences of physicochemical properties of these Rps and compared the expression profiles of *SlRp* genes at three important stages of oogenesis, we found that most *Rp* genes were highly expressed at PS during oogenesis processes, which emphasized the potential roles of Rps on female germ cell differentiation. Collectively, this study could set foundation for characterizing biological functions of Rps in insect oogenesis, which may further serve as a reference for innovative pest control.

## Data Availability

The datasets presented in this study can be found in online repositories. The names of the repository/repositories and accession number(s) can be found in the article/Supplementary Material.

## Supplementary Material

Supplementary Figure S1 Phylogenetic analysis of *Sl*Rps with other five insects. *Bm*, *B. mori*; *Sf*, *S. frugiperda*; *Ha*, *H. armigera*; *Px P. xylostella*; *Dm*, *D. melanogaster*. The phylogenetic NJ tree was constructed using the MEGA X software with the neighbor-joining method. The bootstrap values are 1000 replicates.

Supplementary Figure S2 A network diagram of the regulatory relationships between the presumed miRNAs and particular *SlRp* genes. **(A–C)** showed three representative regulation networks for mir-137-2, *RPSA* and *RPS25*, respectively.
