# Supplementary material for: Genome-Wide Identification and Stage-Specific Expression Profile Analysis Reveal the Function of Ribosomal Proteins for Oogenesis of Spodoptera litura
Source: Front Physiol. 2022 Jun 23;13:943205. doi: 10.3389/fphys.2022.943205 (PMC9259932; doi:10.3389/fphys.2022.943205)
Supplement: Supplementary file 2 [file Table1.docx]

Supplemental Table 1 Primer sequences used in this work

| **GenBank** | **Symbol** | **Forward primer (5’→3’)** | **Reverse primer (5’→3’)** | **Length of Product (bp)** |
| --- | --- | --- | --- | --- |
| **Primer sequences for qRT-PCR** | | | | |
| XM_022980543.1 | *GAPDH* | TGATGGACCCTCTGGAAAA | AGCAACAGGAACACGGAAA | 151 |
| XM_022977357.1 | *SlRPL12* | GTTGGTGCCACTTCATCT | TTGCTTCTTGCGGTCA | 222 |
| XM_022959636.1 | *SlRPS9* | TGGATGAGAAGCAGATGA | AGGCGAACAATGAATGA | 193 |
| XM_022965662.1 | *SlRPS23* | AAGCCCCGTGGTATTC | CGGATGGCAGAGTTAGG | 194 |
| XM_022969695.1 | *SlRPL5* | AAAACCGACTACTATGCCC | CAAACCAACCTTGACGC | 198 |
| XM_022967060.1 | *SlRPS8* | CGCCCATCCGTAAGA | ACTCGTTGTTGGAAGCA | 213 |
| XM_022969890.1 | *SlRPL8* | TCCTGATGCCAAGCG | AACGGGGTTCATAGCC | 202 |
| XM_022971295.1 | *SlRPL10A* | ATGAGGCTAAGACTCTGACG | TGGAACTTGATGGTGGC | 250 |
| XM_022968776.1 | *SlRPLP0* | TCATCCCTGCCCACA | GGAATCGTAGACCTGCTTA | 221 |
| XM_022974894.1 | *SlRPL4* | CCTTCCGCAACATCC | AGACGGCTCAGGTCAGT | 217 |
| XM_022959178.1 | *SlRPL9* | GCGAGTCCTGAAGGTAGA | CAGTTGATGGGGAAATG | 153 |
| XM_022959223.1 | *SlRPL40* | CAAAGATCCAGGATAAAGAG | ATACGGAGGGAAGGCT | 169 |
| XM_022962014.1 | *SlRPS25* | GCAGCCCCAGAAAACA | ACCTCGGACCTTCAACC | 205 |
| XM_022962751.1 | *SlRPS7* | AGGCTGGTGCGTGAA | CCAAGATGGCGTCGTA | 160 |
| XM_022962473.1 | *SlRPS12* | GCTTGCTGAGAACTGCG | GCCGACGATTTTCCTG | 163 |
| XM_022962472.1 | *SlRPL7* | CGCAAGAGGAGGGAA | ACGGATACGGATGACG | 225 |
| XM_022963351.1 | *SlRPL32* | CTACCCAATGGTTTCCG | GGTGACCCTGATGCTGA | 162 |
| XM_022963607.1 | *SlRPL10* | GATGCGTGGTGCTTTC | CCCCATTTCTTGGAGAC | 174 |
| XM_022965074.1 | *SlRPL3* | ATCAAGGGTTGCTGTATGG | TTAAGACTGGGCGGGAG | 246 |
| XM_022965735.1 | *SlRPL19* | ATCAAGAAGCCTGTAGCG | GGAGCAGTTTACGGAGAA | 169 |
| XM_022966303.1 | *SlRPL13* | ATGGGGAAGGGAAATAA | CAACGGGTCTCAAAGGT | 172 |
| XM_022970499.1 | *SlRPL7A* | CGTGCCCTACTGTATTGTC | CTCGTTGAAGTTAGTCTTGATG | 151 |
| XM_022971369.1 | *SlRPS5* | AGCCATCTGGTTGTTGTG | GTTGGACTTAGCGACACG | 163 |
| XM_022972415.1 | *SlRPL30* | CTCCGTCAAGGCAAAGC | CAGAATCACCAGGGTCAGTA | 196 |
| XM_022972524.1 | *SlRPLP1* | ATGTTGCCGTAACTGGTG | TTCCTCCTTCTTCTTCTCCT | 230 |
| XM_022972458.1 | *SlRPL35Aa* | GACGCAGACTTCTACGCT | CCTGGAGGGATACAACATA | 225 |
| XM_022972450.1 | *SlRPL35Ab* | GACGCAGACTTCTACGCT | CCTGGAGGGATACAACATA | 225 |
| XM_022981672.1 | *SlRPS11* | ATCCGTGGTCGTATCCTC | TTCGTGACTTTGAGTACGTTAAAAC | 244 |
| XM_022958370.1 | *SlRPL27* | CGAAGGCACCTCAGACA | GTAGCGAGTGGGCATCA | 166 |
| XM_022958526.1 | *SlRPL36A* | AGGTTATGGTGGTCAATCC | CATCTGTCCCTTCCTCTTC | 166 |
| XM_022960936.1 | *SlRPL37* | GGGTACTTCGAGCTTTGG | GCCTCCTCACGATTTTCA | 209 |
| XM_022961496.1 | *SlRPL23* | AGTTCCGCATCTCGTTG | TCTGGTTTACCCTTCTTGAC | 175 |
| XM_022962124.1 | *SlRPS29* | GGGTCACGCTAACATTTG | AGCTTCTTGAAACCGATG | 162 |
| XM_022962911.1 | *SlRPS20* | ACCCTGACCTCCCGTAA | GAAGTGATCTGCTTCACAATC | 242 |
| XM_022964289.1 | *SlRPS15A* | GCCTTGTTCCAAAGTTATCG | TTGTGAGGACCAGGTAGCC | 230 |
| XM_022964186.1 | *SlRPL23A* | ACAAGTTGTTAAACCCGTTGC | GCTGCTTCAGATGTCAGAGGATA | 222 |
| XM_022964932.1 | *SlRPL35* | ATACGGGTGGTGAGGAA | TAAACACGAGGAGGGAAA | 218 |
| XM_022965365.1 | *SlRPS18* | AGAAGAGGTGGAAAAGATTG | CCCCAGTAGTGACGCATA | 201 |
| XM_022967067.1 | *SlRPL37A* | CCAAACGCACGAAGAAG | AGAATACCCACGCACCA | 210 |
| XM_022966877.1 | *SlRPL13A* | CTCCGTGTCTTCTGCTTG | CGTCTTCGCAACCTTGTCGCCGGAT | 200 |
| XM_022967086.1 | *SlRPL11* | GTGACAACTTCTCAGCCACT | TGCCAGTCTTGCGTCTT | 162 |
| XM_022967506.1 | *SlRPS26* | ACGTTAAGGCTGTGAGATG | AACTTTGCTGTGAATGGC | 200 |
| XM_022967393.1 | *SlRPL34* | ACCAAATCAAACCAGAGGA | CACTTGTGGCAAAGAACAC | 209 |
| XM_022967912.1 | *SlRPS3* | CGGAGACGGAGTTTTCA | ACAGACTGCTCGGGGAT | 216 |
| XM_022967979.1 | *SlRPS16* | AGGTGCGTGACGATTTG | TCCTTGCCGAGCAGTAG | 202 |
| XM_022968395.1 | *SlRPS3Aa* | ATCGCTTCTGAGGGTTTG | TATCCGTCCGTGGTCTTCACGTCAA | 242 |
| XM_022969052.1 | *SlRPL17* | GCCTGATAACCCAGCGAAGT | CGTTGAAGCGGCGGAAT | 173 |
| XM_022969017.1 | *SlRPS17* | GACTAAGACCGTCAAGAAAGC | CTCATCAAATGGGTAGCAAA | 162 |
| XM_022969013.1 | *SlRPS15* | ATTTGCCCGTGGTCTGA | AAAAGTCTTGCCGTTGTAG | 175 |
| XM_022968889.1 | *SlRPS6* | GCAAGTCTGTCCGTGGTT | TCTGGGTTTGGCGTTCT | 242 |
| XM_022968742.1 | *SlRPL36* | GTCTCCAAACCAAGCACTC | GGCAGCCTTCCTCATCT | 212 |
| XM_022969888.1 | *SlRPL24b* | TAAGGTGGACGGCAAGA | ATGGCACGCTGGTATTT | 180 |
| XM_022969828.1 | *SlRPS14* | GTGAGACAGTGTTCGGAGTAG | GTGGCACGCAGTTTGAT | 229 |
| XM_022970356.1 | *SlRPL15* | GTTATGCGTTTCCTCTTGC | GGTTTACACCCTGGCTCTT | 220 |
| XM_022970948.1 | *SlRPS19* | TGAAGAAGACGGGTAAGG | CGAAGATTTTGGTGACAGT | 174 |
| XM_022972238.1 | *SlRPL18A* | ATGAGTCTCGGTCTGGTGT | GCTGTTGTGGAACTGCTT | 194 |
| XM_022972513.1 | *SlRPS30* | TGGGCAGGAGACGATAG | GGGAACCGTGGACTTTAC | 185 |
| XM_022972122.1 | *SlRPS27A* | TAGCCGTACTTCGGTTCT | GTCTATCGTCGTCCTTGAA | 168 |
| XM_022972107.1 | *SlRPS13* | CTGACCGCTGACGATGT | CTTCCTGTTGCGTTCCA | 240 |
| XM_022973155.1 | *SlRPS10* | AAATCGTACCCGCAACC | TCGTCCGAAACCTCCTC | 203 |
| XM_022973774.1 | *SlRPS2* | AGGTTACTGGGGTAACAAGA | CAAAGTTTCCGAGGGTG | 209 |
| XM_022973850.1 | *SlRPL31* | TCTGCAAGCCCTTGATT | GAGGCTACGGGTACGTAGGTGACGA | 166 |
| XM_022973871.1 | *SlRPL21* | TTGTTTGCCCGCAGATT | TGCCCAGAGCGTGTTGT | 181 |
| XM_022973884.1 | *SlRPL27A* | GCCTCAAGTACGCCACA | AAAACGCAGACTCCTCCTA | 178 |
| XM_022974013.1 | *SlRPL14* | GGAAAGTTAGTAAGCGTGGTC | TTGGCGAGTTTTGTGGC | 227 |
| XM_022975139.1 | *SlRPS24* | TGTTCGTATTCGGCTTCA | ACGCACCTTCTTCATCCT | 191 |
| XM_022975495.1 | *SlRPL22* | GAAACCCGTGGCTAAAA | CTTGTCCCTGGCGATGA | 244 |
| XM_022975206.1 | *SlRPSA* | TCTCCCCTCCGTTTCGT | AGCCTGTTGCTCCTCCTTC | 198 |
| XM_022975454.1 | *SlRPL29* | CCACAGGAATGGCATCA | TTTCTTAGCCTTGGCTTCT | 172 |
| XM_022975848.1 | *SlRPL6* | GTGAGAAGAATGGAGGAACC | TGACGAGCAACAGACCG | 228 |
| XM_022975968.1 | *SlRPLP2* | CTCAATGGCAAGAGCGTT | TAGTCAAACAGACCGAAGC | 203 |
| XM_022978257.1 | *SlRPS27* | GTGCCACATCCGAACTC | CTCCTAAACGAACATCCCT | 170 |
| XM_022979273.1 | *SlRPL38* | GAAGGCACGCAGGAAAG | ACCTGGAGACCTGGTGGTA | 159 |
| XM_022979476.1 | *SlRPL26* | AGTTCAGGTTGTCCGTGGTC | CGGTCCTTGTTCATCTTTAGTT | 183 |
| XM_022980543.1 | *SlRPS4* | CAAGGTGGACGGTAAAGTG | CGGGGTAACGGATGGTA | 242 |
| XM_022961256.1 | *SlRPS21* | CAGCAACCGTCTGATCC | CGTCCTTCTTGGTGAGC | 173 |
| XM_022966982.1 | *SlRPS3Ab* | ATCGCTTCTGAGGGTTTG | AGGTATCCGTCCGTGGT | 242 |
| XM_022973872.1 | *SlRPL39* | ATGTCGGCACACAA | TTACAACTTGAGCTTAGT | 156 |
| XM_022974682.1 | *SlRPS28b* | CGTTCTTGCTCGTGTTGT | ACGCACTGGTCCTTTTAC | 127 |
| XM_022975303.1 | *SlRPL24a* | TAAGGTGGACGGCAAGA | ATGGCACGCTGGTATTT | 180 |
| XM_022979129.1 | *SlRPS28a* | CGTTCTTGCTCGTGTTGT | TTCTTATGATCTGACGGCTA | 107 |
| XM_022980942.1 | *SlRPL28* | GGCTAAGGCGACAAACAAGC | GGCTAAGGCGACAAACAAGC | 175 |
| **Primer sequences for RNAi** | | | | |
| XM_022968776.1 | Ds*RPLP0* | *TAATACGACTCACTATAGG*AATGTTTCATCGTGGGTG | *TAATACGACTCACTATAGG*AATGGAGTGAGGAGCAGAG |  |
| XM_022962751.1 | Ds*RPL7* | *TAATACGACTCACTATAGG*GTGAAGGGTAAGGAGGACA | *TAATACGACTCACTATAGG*CACTCTTCAGGTTGGGGTA |  |
| XM_022968889.1 | Ds*RPS6* | *TAATACGACTCACTATAGG*AGGGAGTCCTCACCAACA | *TAATACGACTCACTATAGG*TGCGAGCAACTTAGCGTA |  |
| XM_022965735.1 | Ds*RPL19* | *TAATACGACTCACTATAGG*AAGAAAGTATGGCTTGATCC | *TAATACGACTCACTATAGG*GCCTCCTTGACCTTGTTAC |  |
