# Supplementary figures and images for: Genome-Wide Identification and Stage-Specific Expression Profile Analysis Reveal the Function of Ribosomal Proteins for Oogenesis of Spodoptera litura
Source: Front Physiol. 2022 Jun 23;13:943205. doi: 10.3389/fphys.2022.943205 (PMC9259932; doi:10.3389/fphys.2022.943205)

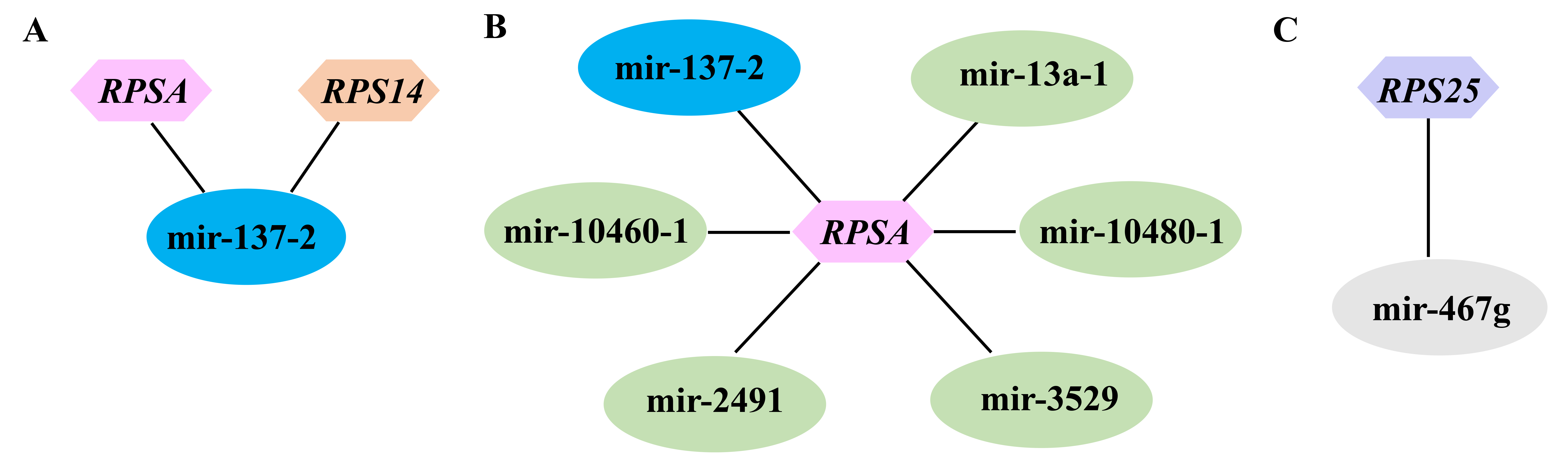

Supplement: Supplementary file 5 [file Image2.TIF]

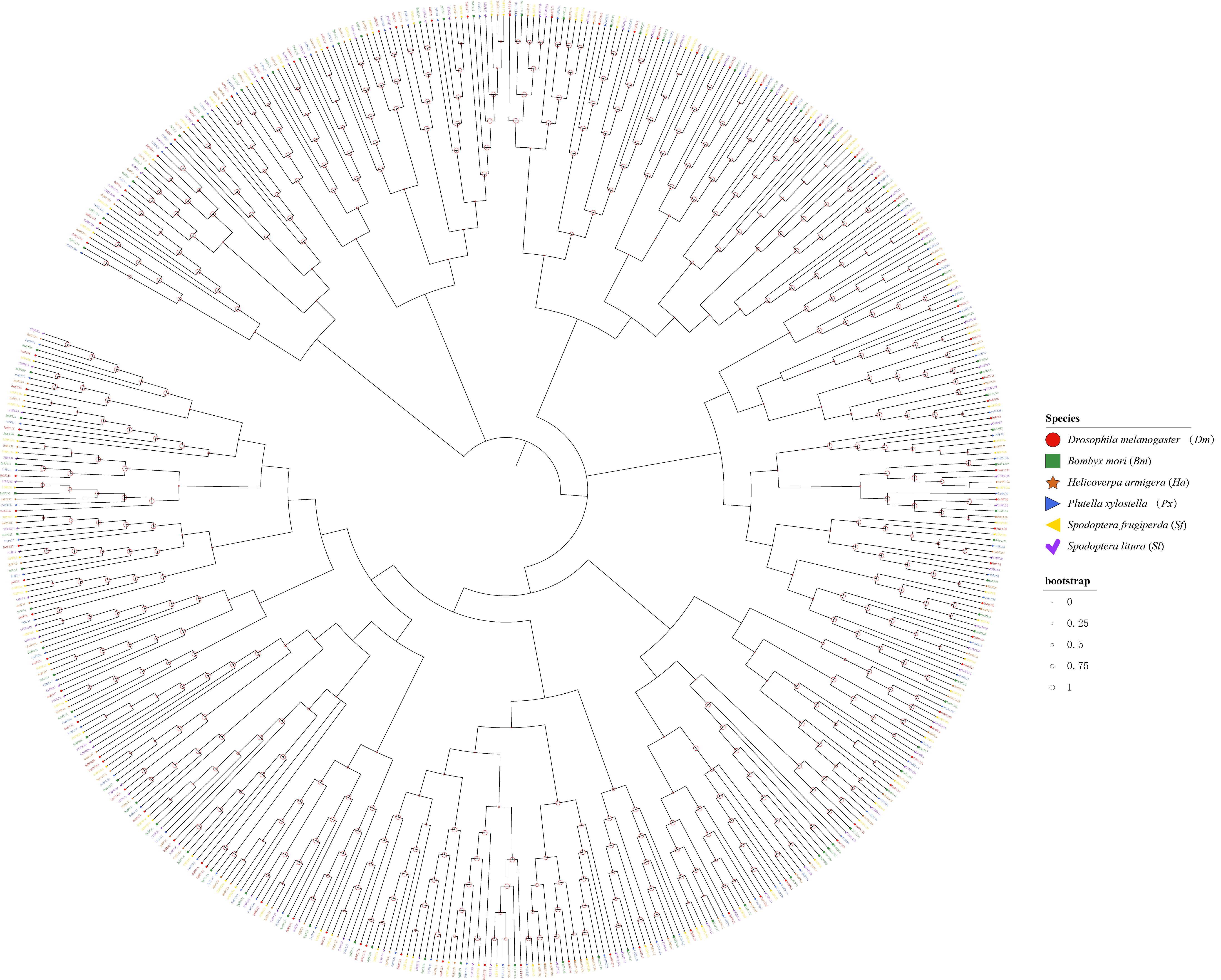

Supplement: Supplementary file 6 [file Image1.TIF]
